# Supplementary material for: Objective QC for diffusion MRI data: Artefact detection using normative modelling
Source: Imaging Neurosci (Camb). 2024 Apr 26;2:imag-2-00144. doi: 10.1162/imag_a_00144 (PMC12247611; doi:10.1162/imag_a_00144)
Supplement: Supplementary Tables [file imag_a_00144-supp.pdf]

Supplement 1

Table S1: List of quantitative dMRI QC metrics obtained using automated QC methods.

**QC metric**	**Description**	**Source**
Average absolute motion (mm)	Volume-to-volume motion	EDDY QC
Average relative motion (mm)	Volume-to-volume motion	EDDY QC
Average x translation (mm)	Volume-to-volume motion	EDDY QC
Average y translation (mm)	Volume-to-volume motion	EDDY QC
Average z translation (mm)	Volume-to-volume motion	EDDY QC
Average x rotation (deg)	Volume-to-volume motion	EDDY QC
Average y rotation (deg)	Volume-to-volume motion	EDDY QC
Average z rotation (deg)	Volume-to-volume motion	EDDY QC
Average stdev x translation (mm)	Within volume motion	EDDY QC
Average stdev y translation (mm)	Within volume motion	EDDY QC
Average stdev z translation (mm)	Within volume motion	EDDY QC
Average stdev x rotation (deg)	Within volume motion	EDDY QC
Average stdev y rotation (deg)	Within volume motion	EDDY QC
Average stdev z rotation (deg)	Within volume motion	EDDY QC
Stdev x axis linear term (Hz/mm)	Eddy current-induced distortion	EDDY QC
Stdev y axis linear term (Hz/mm)	Eddy current-induced distortion	EDDY QC
Stdev z axis linear term (Hz/mm)	Eddy current-induced distortion	EDDY QC
Total number of outliers (%)	Slice drop-out	EDDY QC
Volume-wise number of outliers (%)	Slice drop-out	EDDY QC
Outliers per b-shell (%)	Slice drop-out	EDDY QC
Outliers per PE dir (%)	Slice drop-out	EDDY QC
Average b0 SNR	General scan quality	EDDY QC
Average diffusion CNR per shell	General scan quality	EDDY QC
Number of slices corrected	Slice drop-out	UK Biobank
T1 vs dMRI discrepancy	Movement artefacts	UK Biobank
FA discrepancy	Movement artefacts	Locally derived
